# Comparative Evaluation of Mogibacterium timidum in the Subgingival Plaque of Periodontally Healthy and Chronic Periodontitis Patients: A Real-Time Polymerase Chain Reaction (PCR) Study

**DOI:** 10.7759/cureus.61211

**Published:** 2024-05-28

**Authors:** Vangmayee Shikarkhane, Vidya Dodwad, Swapna A Patankar, Pooja Pharne, Nishita Bhosale, Amod Patankar

**Affiliations:** 1 Periodontology, Bharati Vidyapeeth (Deemed to be University) Dental College and Hospital, Pune, IND; 2 Oral Pathology and Microbiology, Bharati Vidyapeeth (Deemed to be University) Dental College and Hospital, Pune, IND; 3 Oral and Maxillofacial Surgery, Bharati Vidyapeeth (Deemed to be University) Dental College and Hospital, Pune, IND

**Keywords:** real-time pcr testing, subgingival plaque, anaerobic bacteria, chronic periodontitis, mogibacterium timidum

## Abstract

Background: *Mogibacterium timidum* is a new genus of anaerobic bacteria discovered in the year 2000. It is one of the most common bacteria present in the host microbial flora of dental plaque. The levels of *M. timidum* are supposedly higher in inflammatory conditions.

Aims and objectives: This study aimed to quantify the levels of *M. timidum* species in the subgingival plaque samples of healthy patients and patients with chronic periodontitis.

Materials and methods: A total of 24 samples of the subgingival plaque, 12 healthy samples and 12 samples of chronic periodontitis patients, were collected in a buffer solution using a sterile Gracey curette. These samples were then sent to a laboratory for real-time polymerase chain reaction (PCR) testing.

Results: *M. timidum* was found in higher quantities in plaque samples taken from chronic periodontitis patients when compared to healthy patients.

Conclusion: *M. timidum* can be said to be associated with chronic periodontitis condition. Further studies are required to know the exact nature of the pathogen.

## Introduction

The human oral cavity has an abundance of microorganisms. A healthy state is seen in the oral cavity if these microorganisms are indigenous. These are non-pathogenic and have no role in disease initiation or progression. Microbes from phyla *Firmicutes*, *Proteobacteria*, *Bacteroidetes*, *Actinobacteria*, *Fusobacteriota*, and *Pseudomonadota* are indigenous. Gingivitis and periodontitis are common diseases that manifest as local inflammation affecting the periodontium. Gingivitis is a non-destructive disease but, if left untreated, may lead to periodontitis. Lack of maintenance of oral hygiene will make a suitable environment for bacteria to initiate gingivitis and progress to periodontitis [1].

According to various studies, more than 600 species of bacteria house the human subgingival plaque biofilm [2]. A new genus *Mogibacterium* was put forth in the year 2000, and some unique species belonging to the same genus were included in it [3]. They are characterized as asaccharolytic and strictly anaerobic Gram-positive rod-shaped bacteria. In any broth media, an extremely poor growth is exhibited by these cells which are non-sporing and non-motile in nature. Formerly known as *Eubacterium timidum*, *Mogibacterium timidum* has been isolated from various sites in the body where inflammatory conditions were present [4]. This bacterium has been isolated mainly from patients with Ludwig's angina, head abscesses, and acute lung and liver infections [5].

This bacterium has also been found in the subgingival biofilm and deep periodontal pockets of chronic periodontitis patients [6]. Also, with increasing severity of gingivitis, an increase is seen in *M. timidum* levels suggesting a high correlation between this bacterium and gingivitis and periodontitis. This study, therefore, aims to evaluate and compare levels of *M. timidum* in the subgingival plaque of periodontally healthy and chronic periodontitis patients.

## Materials and methods

Twenty-four subjects, 12 subjects, healthy periodontally, and 12 subjects with chronic periodontitis who reported to the outpatient department of Periodontology of Bharati Vidyapeeth (Deemed to be University) Dental College and Hospital, Pune, Maharashtra, were selected. All eligible subjects were explained in detail about the potential risks involved if any, the manner in which the study will be conducted, and the benefits the participants will avail in the study, and they were asked to sign the informed consent. Approval for the study was obtained from the Institutional Ethics Committee of Bharati Vidyapeeth (Deemed to be University) Dental College and Hospital (approval number: EC/NEW/INST/2021/MH/0029).

The inclusion criteria for periodontally healthy subjects (Group A) were subjects willing to participate in the study with written informed consent, subjects showing no clinical or radiographic signs of periodontitis, systemically healthy subjects, sites with the presence of subgingival plaque and bleeding on probing, and subjects from age 30 years onwards.

The inclusion criteria for chronic periodontitis subjects (Group B) were subjects willing to participate in the study with written informed consent, systemically healthy subjects, at least 20% of the sites showing clinical attachment levels and probing depth ≥5mm and bleeding on probing, and subjects from age 30 years onwards.

The exclusion criteria for both Groups A and B were subjects who are medically compromised, subjects who have received surgical periodontal treatment in the last six months, pregnant, lactating women, subjects with antibiotic therapy in the previous six months, and subjects with smoking and tobacco chewing habits and prolonged administration and use of inflammatory and immunosuppressive drugs.

Clinical parameters

All teeth other than the third molars were included. The following parameters were examined clinically at the mesiolingual, lingual, distolingual, mesiobuccal, buccal, and distobuccal sites using a periodontal probe (UNC-15) manually: gingival index by Loe and Silness, plaque index (Turesky-Gilmore-Glickman modification of the Quigley-Hein plaque index), pocket probing depth, and clinical attachment level.

Prior to collecting the samples, the plaque present supragingivally was cleaned using sterilized cotton pellets, and cotton rolls were used to isolate the sites to facilitate the collection of subgingival plaque without contamination. 

Subgingival plaque samples were collected from the gingival sulcus in healthy subjects and from deep pockets (PD >5mm) per subject in chronic periodontitis subjects using a sterile Gracey curette. Subgingival plaque samples were later then transferred in 1.5 ml microcentrifuge tubes having tris-ethylenediaminetetraacetic acid buffer (TE buffer). The samples were further processed for DNA extraction using the modified proteinase K method.

DNA extraction

Briefly, samples containing subgingival plaque were flushed with fresh TE buffer three times. Then 50 µl of lysis buffer I (1% Triton X-100, Tris-HCL pH 8.0 10 mM, and EDTA 1 mM) was added followed by 50 μl of lysis buffer II (Tris-HCl pH 8.0 50 mM, KCL 50 mM, MgCl2 2.5 mM, Tween-20 0.45%, and Nonidet P-40 0.45%). For the degradation of proteins, proteinase K (10 mg/ml) was then added. The samples were incubated at 60°C for two hours and then in boiling water bath for 10 minutes. Purification of DNA was done by using absolute ethanol and 3M sodium acetate. The DNA bolus was then broken down in molecular-grade water and kept at -20°C till any use [7].

The quality and purity of DNA samples were confirmed by using a biophotometer.

Real-time PCR

A realplex Mastercycler (Eppendorf, Hamburg Germany) having a 95-well format was used for conducting real-time qPCR along with amplification and detection. Reactions were established in a laminar airflow to control any possible contamination. The primers targeting the species-specific sequence of the 16S ribosomal ribonucleic acid gene of *M. timidum* were used in the study: forward primer 5ʹ-AAGCTTGGAAATGACGC-3ʹ and reverse primer being 5ʹ-CCTTGCGCTTAGGTAA-3ʹ. The procedure was done with a total volume of 20 µl in 0.2 ml clear cap tube strips. TB Green Premix Ex Taq (Tli RNaseH Plus) PCR master mix was used in the reaction mixture which contained TaKaRa Ex Taq HS, dNTP mixture, Mg2+, Tli RNase H, and TB green dye. DNA template and primers were added at optimum concentrations. The tubes were then kept in a real-time PCR Mastercycler to run different temperature cycles. Activation of the enzyme was carried out at 95°C for three minutes, and then denaturation was done in the form of 40 cycles at 95°C for 20 seconds, annealing at 50-60°C for 20 seconds, and extension at 72°C for 20 seconds [8].

Statistical analysis

All analyses were carried out using IBM SPSS Statistics for Windows, Version 25.0 (Released 2017; IBM Corp., Armonk, New York, United States). Detailed statistics were expressed as means and standard deviation and numbers and percentages. Intergroup comparison between the *M. timidum* detection frequency was done using the chi-squared test. In the above test, a p-value less than or equal to 0.05 was considered to be statistically significant.

## Results

In Group A, two males and 10 females in the range of 30-45 years completed the study, whereas in Group B, five males and seven females in the range of 30-40 years completed the study.

Table 1 shows the distribution of age and gender in the study population. No difference was observed in the average age of participants in both groups. It was observed that the number of female participants was higher than male participants.

**Table 1:** Distribution of age and gender in study participants Data for age is represented as mean±SD. Data for gender is represented as N, %

	Age (mean±SD)	Gender (N, %)	
		Males	Females
Group A (healthy periodontium) (N=12)	36.33±5.12	2 (16.7)	10 (83.3)
Group B (chronic periodontitis) (N=12)	36.00±4.45	5 (41.7)	7 (58.3)

Table 2 shows the parameters measured clinically. 

**Table 2:** Full mouth clinical parameters of study participants Data for GI, PI, PD, and CAL is represented as mean±SD GI: gingival index; PI: plaque index; PD: probing depth; CAL: clinical attachment level

	GI (%)	PI (%)	PD (mm)	CAL (mm)
Group A (healthy periodontium) (N=12)	20.2±14.5	22.9±18.7	2.3±4.6	1.4±3.6
Group B (chronic periodontitis) (N=12)	59.6±29.5	65.8±32.7	4.8±2.5	3.6±2.8

Table 3 shows the detection frequency of *M. timidum* in both groups. *M. timidum* was detected in 66.66% of chronic periodontitis patients, whereas it was detected in 33.33% of healthy individuals.

**Table 3:** Comparison of the detection frequency of Mogibacterium timidum between the two study groups Data for the frequency of *Mogibacterium *detection is represented as N, % p≤0.05 is considered statistically significant

	Number	Percentage
Group A (healthy periodontium) (N=12)	4	33.33%
Group B (chronic periodontitis) (N=12)	8	66.66%
P-value (chi-squared test)	0.289	

**Figure 1:**
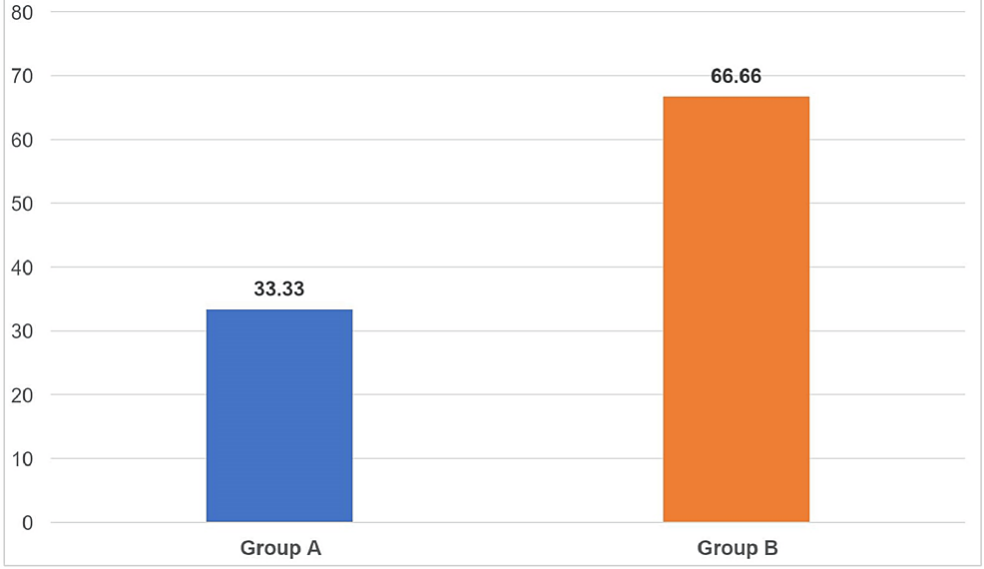
Comparison of the frequency of detection of Mogibacterium timidum between the two study groups Data for the frequency of *Mogibacterium *detection is represented as %

Figure 1 shows the comparison of the frequency of detection of *M. timidum* between the two study groups.

## Discussion

*M. timidum* is a strictly anaerobic Gram-positive rod-shaped bacterium. This bacterium is generally found to be associated with inflammatory conditions in the body. Its presence has been confirmed in subgingival plaque present in deep periodontal pockets. In the present study, *M. timidum* is highly detected in subjects suffering from chronic periodontitis than in periodontally healthy subjects. Its association with any systemic disease cannot be confirmed as systemically healthy subjects are included in both groups.

Different techniques have been used in the past to detect the presence of *M. timidum* in various samples from the oral cavity. *M. timidum* has been found in head and neck infections [9] like Ludwig's angina, chronic periodontitis [1], and endodontic infections [8] using the species-specific PCR method. 16S rDNA clone library analysis containing detection primers of broad-spectrum and checkerboard DNA-DNA hybridization are some other methods to detect this organism.

*M. timidum* was first isolated in deep periodontal pockets by Holdeman et al. in 1980 [4]. In further studies conducted by Moore et al. [10,11], a strong association was found between *M. timidum* and periodontitis. In the initial study conducted, *M. timidum* was isolated in periodontal pockets in 70% (N=90) of chronic periodontitis among the affected population. This bacterium has been frequently detected in older individuals as compared to younger individuals according to an experimentally induced gingivitis model. In individuals with worsened signs of gingivitis, *M. timidum* was detected with a higher frequency [10]. Later, Moore et al. [6] detected 70% and 11% *M. timidum* levels in severe chronic periodontitis and healthy patients. These present results confirm the findings of the existing study and affirm the role of *M. timidum* in increasing the probability of gingivitis progressing to periodontitis and the increased susceptibility to experimental gingivitis.

Similar studies on *M. timidum* have been conducted to evaluate its role in the etiology of periodontitis. In 2004, Booth et al. [12] confirmed that *M. timidum* is present in shallow pockets in patients with chronic periodontitis using the slot-blot hybridization technique. Mayanagi et al. [1] conducted a study where *M. timidum* bacterium was evaluated in supragingival and subgingival plaque from healthy and chronic periodontitis subjects. In supragingival plaque, *M. timidum* was seen in 15% of healthy subjects and in 65% of chronic periodontitis subjects. In subgingival plaque, it was found in 10% of healthy subjects and 70% of chronic periodontitis subjects. Colombo et al. [13] stated that patients with chronic periodontitis showing increased levels of *M. timidum* exhibited periodontitis of the refractory type which results in significantly less rise of clinical attachment levels. According to these studies, there is an association between *M. timidum* and periodontitis.

*M. timidum* is one of the many microorganisms found in the oral cavity. In the present study, subgingival plaque taken from healthy sites showed negligible quantities of *M. timidum*. Significant amounts of bacteria have been detected in plaque collected from deep periodontal pockets in patients where increased periodontal destruction was seen. This finding supports the role of *M. timidum* in causing the breakdown of periodontium. However, the role of other periodontal pathogens also cannot be neglected. Considering the significance of these results, studies need to be carried out in the future to analyze the patterns of virulence and the molecular patterns associated with the pathogenicity of this species.

Limitations of the study

This study was conducted using a small sample size, and also, no follow-up was conducted after scaling and root planing to assess the effect of mechanical debridement in reducing the bacterial levels. The exact role of *M. timidum* could not be established in the pathogenesis of chronic periodontitis.

## Conclusions

Our study critically evaluates and compares the levels of *M. timidum* in the subgingival plaque of healthy and individuals with chronic periodontitis. Inflammatory lesions in the body show the presence of *M. timidum*. Many other pathogens are also associated with the pathogenesis of periodontitis. Knowledge regarding specific microorganisms such as *M. timidum* is important to establish the relation of the role the organism has in the initiation and progression of periodontitis. Different species could influence periodontal destruction, and further research and analysis are required about *M. timidum*.
